# Analysis of the Influence of Manufacturing Technology on Selected Static, Fatigue and Morphological Properties of CFRP Composites

**DOI:** 10.3390/ma18010102

**Published:** 2024-12-30

**Authors:** Andrzej Kubit, Kishore Debnath, Ján Slota, Filip Dominik, Ankit Dhar Dubey, Gorrepotu Surya Rao, Krzysztof Żaba

**Affiliations:** 1 Department of Manufacturing and Production Engineering, Rzeszow University of Technology, Al. Powst. Warszawy 8, 35-959 Rzeszow, Poland; 2 Department of Mechanical Engineering, NIT Meghalaya, Shillong 793003, India; debnath.iitr@gmail.com (K.D.); p22me007@nitm.ac.in (A.D.D.); gsuryarao@nitm.ac.in (G.S.R.); 3 Institute of Technology and Materials Engineering, Technical University of Košice, Mäsiarska 74, 040 01 Košice, Slovakia; jan.slota@tuke.sk (J.S.); filip.dominik@tuke.sk (F.D.); 4 Department of Non-Ferrous Metals, AGH University of Science and Technology, 30-059 Krakow, Poland; krzyzaba@agh.edu.pl

**Keywords:** CFRP composites, autoclave, infusion, hand lay-up, vacuum, mechanical testing, NDT, fatigue properties

## Abstract

The aim of this study was to compare the mechanical properties of carbon-fiber-reinforced polymer (CFRP) composites produced using three popular technologies. The tests were performed on composites produced from prepregs in an autoclave, the next variant is composites produced using the infusion method, and the third variant concerns composites produced using the vacuum-assisted hand lay-up method. For each variant, flat plates with dimensions of 1000 mm × 1000 mm were produced while maintaining similar material properties and fabric arrangement configuration. Samples for testing were cut using a plotter in the 0° and 45° directions. Non-destructive tests (NDTs) were carried out using the active thermography method, demonstrating the correctness of the composites, i.e., the absence of structural defects for all variants. Static peel strength tests were carried out for samples with different directional orientations. The tests were carried out at temperatures of +25 °C and +80 °C. At room temperature, similar strengths were demonstrated, which for the 0° orientation were 619 MPa, 599 MPa and 536 MPa for the autoclave, vacuum and infusion variants, respectively. However, at a temperature of +80 °C, only the composite produced in the autoclave maintained the stability of its properties, showing a strength of 668 MPa. Meanwhile, in the case of the composite produced by the infusion method, a decrease in strength at an elevated temperature of 46.5% was demonstrated, while for the composite produced by the hand lay-up method, there was a decrease of 46.2%. For the last two variants, differential scanning calorimetry (DSC) analysis of epoxy resins constituting the composite matrices was carried out, showing a glass transition temperature value of 49.91 °C for the infusion variant and 56.07 °C for the vacuum variant. In the three-point static bending test, the highest strength was also demonstrated for the 0ᵒ orientation, and the bending strength was 1088 MPa for the autoclave variant, 634 MPa for the infusion variant and 547 MPa for the vacuum variant. The fatigue strength tests in tension at 80% of the static strength for the infusion variant showed an average fatigue life of 678.788 × 10^3^ cycles for the autoclave variant, 176.620 × 10^3^ cycles for the vacuum variant and 159.539 × 10^3^ cycles for the infusion variant.

## 1. Introduction

The remarkable strength-to-weight ratio of CFRP makes it an appealing engineering material. Its specific stiffness and specific strength, which measure relative strength to weight, are higher than those of several common engineered materials, such as polymers, metals/alloys and foams. In comparison to more traditional materials, carbon-fiber-reinforced composites offer significant benefits, including superior strength, durability, light weight, chemical resistance and temperature tolerance [1,2]. Other fiber-reinforced composites such as glass-fiber-reinforced polymer composites provide cost-effectiveness and good toughness properties, but they have lower strength and stiffness in comparison to CFRP [3]. In the case of matrix, thermoplastic resins such as polypropylene provide good toughness and long-term durability whereas thermosetting polymers like epoxy resins offer excellent mechanical properties and thermal resistance [4]. Numerous sectors make extensive use of carbon-fiber-reinforced composites, including construction, transportation, aviation, defense and sports gear [5]. Due to their general applications in sectors where they are subjected to strong and repetitive forces, CFRPs are prone to experiencing damage, particularly in the form of delamination, fiber breakage and microcracking [6]. Air voids and surface irregularities are examples of defects that can be created during production of CFRP composites [7]. In addition, there are still a number of issues that carbon fiber composites face in the field, such as hailstones, landing strip debris, ground equipment collisions, tool drops and bird strikes. Surface dents, delamination, matrix cracking, fiber breaking, wrinkling and waviness are some of the forms of common defects to occur in resin-rich composites during the manufacturing process [8]. But these types of damage are typically invisible to the human eye and thus detection of these types of damage requires sophisticated techniques. These types of damage often occur on the internal surface and are not evident right away. This highlights the significance of conducting thorough inspections of composite structures on a regular basis to ensure their integrity. Non-destructive testing is a useful method to detect defects without damaging the composites and thus is highly recommended for defect detection and evaluation. Thermography is one such technique that has emerged rapidly over the years in the field of defect detection and has several advantages such wide range of observation, wide-area coverage and non-contact detection [9]. Yang et al. [6] used the ultrasonic infrared thermography technique for detection and inspection of defects in CFRP composites for aerospace applications. Li et al. [10] addressed the possibilities of the damage’s occurrence and discussed the detection of damage in CFRP composites through a microwave process aligned with an NDT.

The damage and defects experienced by fabricated composites depend very much on the method of fabrication used. Currently, there are numerous methods used for fabrication of fiber-reinforced composites such as the hand lay-up method, resin transfer molding, compression molding, vacuum infusion, autoclave, etc. Wiggers et al. [11] studied the effect of two distinct manufacturing procedures, autoclave vacuum bagging and resin liquid pressurized preg, for fabrication of composite laminates. The resin liquid pressurized preg process produced laminates with lower mechanical performance than the autoclave method. A larger resin content, increased thickness and non-homogeneous resin peculation contributed to the reduction in mechanical characteristics. Park et al. [12] presented possibilities for enhancing the performance of carbon fiber/epoxy-based composite parts and identified efficient techniques for reducing manufacturing-induced flaws, including internal voids and surface porosity. The authors reported that achieving high-quality parts with a low void content of 1.3% and a high fiber volume fraction of 53% may be possible with improved resin flow. Chokka et al. [13] investigated the tensile and wear behavior of the carbon fiber/epoxy-based composites fabricated by vacuum infusion and the hand lay-up process. The tensile strength of the CFRP composites produced through vacuum infusion was higher, meanwhile, they exhibited inadequate wear resistance. Sha et al. [14] investigated the mechanical properties of a vacuum-assisted resin infusion method for creating low-porosity carbon fiber/phenolic resin composites using a low-viscosity and phenolic resin augmented with ZrO_2_ nanoparticles. In comparison to the autoclave method, the vacuum infusion strategy preserves a higher resin content in composites with increased residual modulus after oxidation. Meier et al. [15] examined the interlaminar void content and shear strength of hexagonal carbon fabric/epoxy composites fabricated by vacuum infusion. The study found that the least amount of void content was found in the composites generated at 10 Hz, whereas the highest percentage was identified in the composites developed at 25 Hz. Selzer and Friedrich [16] explored the impact of moisture on the mechanical characteristics and failure behavior of carbon fiber/epoxy-based composites fabricated by the autoclave process. As moisture was absorbed, the epoxy matrix was softened, and the fiber–matrix adhesion was decreased. Xu and Hoa [17] investigated the mechanical characteristics of nanocomposites composed of carbon fiber/epoxy-based nanocomposites fabricated by the autoclave process. The addition of 2 percent nanoclay raised the interlaminar fracture toughness of the unidirectional carbon-fiber-reinforced composite by 53%, while 4 percent nanoclay increased it by 85%. Elanchezhian et al. [18] examined the mechanical behavior of hand lay-up processed glass/carbon-fiber-epoxy-based composites under different temperature and strain conditions. The tensile load bearing capacity of CFRP composite was 36.262 kN, which was relatively higher than that of GFRP composite. CFRP composite exhibited a flexural loading that was relatively greater than that of GFRP composites, with an ultimate load value of 1.78 kN. Ochola et al. [19] explored the mechanical properties of carbon/glass-fiber-epoxy-based composite by varying the strain rate and observed that as the strain rate increases, the CFRP undergoes complete disintegration.

Dong et al. [20] explored the mechanical properties of carbon-fiber-reinforced epoxy composites by implementing surface modification through carbon black on the carbon fiber and observed that the tensile strength of modified CFRPs exhibited a slight improvement in comparison to that of untreated CFRPs. Chen et al. [21] compared the mechanical properties of carbon fiber/epoxy-based composites fabricated by 3D printing and hand lay-up processes. In comparison to cross-ply specimens generated by the conventional hand lay-up method, the tensile strength and stiffness of carbon fiber composites manufactured using 3D printing were higher. However, the hand lay-up specimens showed higher strength and stiffness under flexural loading. The differences in material characteristics between the two processes of manufacturing indicate each method has its appropriate application. Agarwal et al. [22] examined the effect of fiber loading on the mechanical, thermomechanical and bidirectional properties of carbon fiber/epoxy-based composites fabricated by the hand lay-up process. Bidirectional carbon fiber/epoxy composites exhibited superior mechanical properties compared to short-fiber-reinforced composites. However, the hardness values of short-carbon-fiber-reinforced epoxy composites were superior to those of bidirectional composites. Bergant et al. [23] examined the fatigue and wear behavior of carbon-fiber-reinforced epoxy gears fabricated through the autoclave process and observed enhanced mechanical properties of CFRP gears in comparison to plastic gears. Park et al. [12] compared the autoclave process for high-quality composites with a vacuum bag prepreg method as a cost-effective alternative. The study showed that mechanical properties of vacuum-bag-fabricated composites were equivalent in comparison to autoclave-fabricated composites. Kim et al. [24] fabricated fiber-reinforced composites with the hand lay-up process and vacuum infusion and found that composites with three layers of fibers fabricated with vacuum infusion had better tensile and compressive properties than hand-lay-up-fabricated composites. Abdurohman et al. [25] studied the effect of different fabrication processes such as hand lay-up, vacuum bagging and vacuum-assisted resin infusion on the compressive and shear properties of fiber-reinforced epoxy resin composites and concluded that vacuum-assisted resin-infused composites outperformed other composites in compressive and shear strength by 71% and 53%.

From the above literature survey, it can be observed that there have been multiple works towards the enhancement of mechanical properties of fiber-reinforced epoxy composites and most of the work focused on analyzing the void content in the fabricated composites and their mechanical properties such as fracture toughness, tensile, flexural and compressive properties. Some works also studied the effect of variation of strain rate, surface modification and hybridization on the mechanical properties of GFRPs. Most significantly, all these works have been performed with differing fabrication processes and several works focused on comparison of several fabrication processes.

In summary, there are many research papers available in the literature presenting studies on the mechanical properties of polymer–fiber composites. However, without a doubt, the development of applied technologies and the need to reduce the weight of structures, especially in the aviation and automotive industries, mean that this type of composite is still gaining wider application. Therefore, it is justified to claim that it is still necessary to expand and supplement knowledge in this area. Each study comprehensively comparing the properties of composites manufactured using different technologies is a valuable addition to engineering knowledge. Therefore, this paper aims to briefly compare the properties of composites manufactured using three popular technologies used in the composite industry.

In this study, carbon fiber/epoxy-based composites are developed by using hand lay-up vacuum-assisted (the abbreviated name of this variant used below is: vacuum), vacuum infusion (the abbreviated name of this variant used below is: infusion) and autoclave processes (the abbreviated name of this variant used below is: autoclave). The uniformity and lack of inclusions and voids in the developed composites were examined by NDTs via the active thermography method. The tensile and flexural properties of the developed composites were examined by varying the fabrication method, orientation and testing temperature. The delamination that occurred during the tensile and flexural testing was examined and damage mechanisms were explored by conducting SEM and EDS analyses of the fractured samples of the carbon fiber/epoxy composites.

For selected variants of epoxy resins, DSC analysis was performed, showing the glass transition temperature for the infusion and vacuum variant matrices. Finally, comparative fatigue tests were carried out, determining the fatigue life for individual composite variants at selected levels of cyclic loading. Selected fatigue fractures were subjected to fractographic analyses using SEM.

## 2. Materials and Methods

### 2.1. Fabrication of the Composites

**Figure 1:**
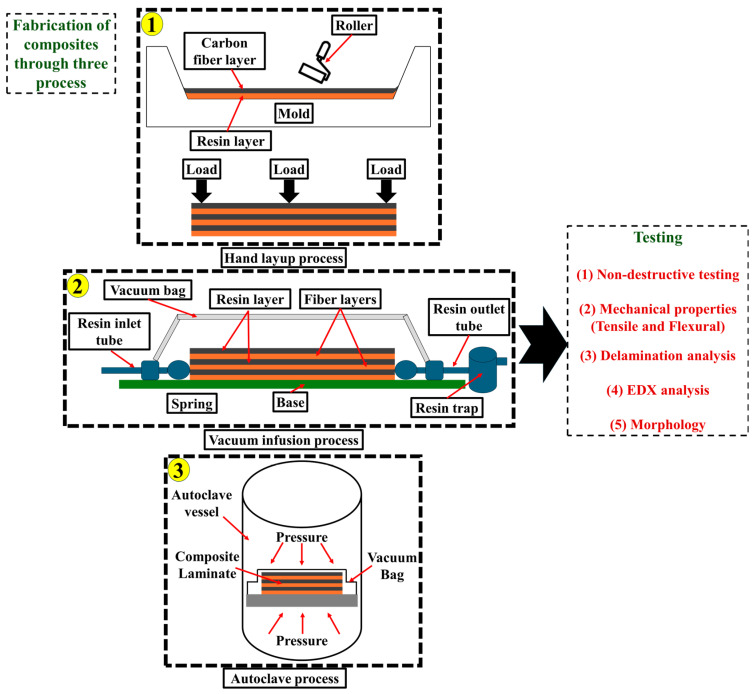
Schematic diagram showing three technologies of fabrication process used for composite specimens; (1) hand layup, (2) vacuum infusion and (3) autoclave process

Epoxy resin and carbon fiber were considered as matrix and reinforcement constituents, respectively, to develop the CF/E composites. Carbon fiber was used in the composite while varying the fiber orientation at 0° and 45°. The CFRP composites were fabricated through three different techniques, namely, hand lay-up vacuum-assisted, infusion and autoclave processes. Figure 1 shows the schematic diagram showing the fabrication processes used in this study.

#### 2.1.1. Fabrication Through Hand Lay-Up

A plain weave carbon fabric with a grammage of 193 g/m^2^ was used. All layers were laid in the same direction. The matrix was made of Havel L285 MGS aerospace-certified epoxy resin with H285 hardener in a weight ratio of 100:40. The lamination was carried out manually by impregnating subsequent layers of fabric with resin, then a vacuum bag was used to suck out excess resin, make the thickness uniform and apply even pressure during curing. The vacuum value was −0.9 bar. Curing was carried out at room temperature (24 h), followed by post-curing at +40 °C for 90 min.

#### 2.1.2. Fabrication Through Vacuum Infusion

In this case, plain weave carbon fabric with a grammage of 193 g/m^2^ was also used. The fabric was laid in the same direction, so the layers were not directional.

A special resin designed for the infusion process with low viscosity (500–900 mPas/25 °C) was used as the matrix. The resin was supplied by Havel Composites, its trade name is LH288, a dedicated hardener H281 was used and the weight ratio was 100:26.

In the process, the reinforcement, i.e., dry carbon fabric, was laid in the mold, then the mold was sealed with foil and butyl tape. The set prepared in this way was subjected to saturation by vacuum suction of the resin, which saturated all the reinforcement layers.

#### 2.1.3. Fabrication Through Autoclave

The plates were made to order by a specialized company producing composite structures by the autoclave process, i.e., Tworzywa Sztuczne PZL Mielec (Mielec, Poland). The plates were made of carbon fiber epoxy prepreg designated HexPly AGP-193 (Hexcel Corporation, Stamford, CT, USA). The laminates were autoclave-cured under the following conditions: heating speed 2 °C/min, curing temperature 177 °C, pressure in autoclave chamber during curing 3 bar, curing time 120 min and cooling speed after curing 3 °C/min. The negative pressure of −0.7 bar in the vacuum bag was maintained until the curing temperature was reached, which took 30 min.

### 2.2. Testing Procedure

The NDT was conducted to verify the quality of composites in terms of uniformity and lack of inclusions and air voids by considering the thermography process by C-CheckIR automation technology. The system includes a C2 IR GigE thermal imaging camera with a built-in modulator for active thermography, a 2 kW halogen lamp and a tablet PC with IR-NDT version 1.7 software configured for testing CF/E composites. The obtained thermogram sequences were analyzed using the discrete Fourier transform. The pulse-phase analysis was implemented during the NDT with the parameters as lamp power: 1600 W, warm-up: 15 s and cooling down: 45 s. Further, mechanical properties of the composite specimen were studied through tensile and flexural testing. The effects of three fabrication methods, the orientation of fibers and testing temperature on the tensile and flexural properties were analyzed. The morphological characteristics of the fractured specimen were further studied through the microscopic images obtained from scanning electron microscopy (SEM). Energy-dispersive X-ray spectroscopy was used to identify and quantify the elements presents in the sample and their distribution in the composite specimen.

The test samples were cut from 1000 mm × 1000 mm plates using a plotter. In order to determine the mechanical properties of the composites in different directions, the strength test samples were cut in two directions, i.e., 0° and 45°.

The static strength tests, both for tensile and three-point bending, were carried out on a Zwick/Roell Z30 (ZwickRoell GmbH & Co.KG, Ulm, Germany) testing machine equipped with a temperature chamber enabling the implementation of tests at elevated temperatures. The tests were carried out at room temperature (25 °C) and at an elevated temperature of +80 °C in order to verify the variability of the properties of composites manufactured using different technologies under the influence of changing operating conditions.

Three-point bending tests were performed for samples measuring 80 mm × 13 mm, with a support span of 64 mm. The tests were conducted at a speed of 2 mm/min. The tests were carried out in accordance with the recommendations of the ASTM-D790 standard. For each tested variant, five repetitions were performed.

Tensile strength tests were performed for samples measuring 250 mm × 15 mm, with the grip part on both sides being 50 mm, hence the measuring length of the samples was 150 mm. In this case, a tensile speed of 5 mm/min was used. The tests were carried out in accordance with the recommendations of the ASTM D3039 standard. For each tested variant, five repetitions were performed.

DSC analysis was performed using a Mettler Toledo 822e calorimeter equipped with StareSystem Version 12.0 software. Analyses were performed in a nitrogen atmosphere (60 mL/min), at a heating rate of 10 °C/min in the range of 0–200 °C. Samples in the form of resin cylinders with a diameter of 10 mm and a height of 6 mm were used for the tests.

Fatigue strength tests of composite specimens were carried out on an HT-9711 Dynamic Testing Machine (Hung Ta Instrument Co., Taichung City, Taiwan). The fatigue tests were carried out at room temperature with a limited number of cycles equal to 2 × 10^6^ and a frequency of 25 Hz, the load ratio of R = 0.1 was used. The same sample dimensions were used as in the static tensile tests and the measuring length was also 150 mm. For each cyclic loading level, four repetitions were performed for samples of each variant.

## 3. Results

### 3.1. Non-Destructive Testing of the CFRP Composites

**Figure 2:**
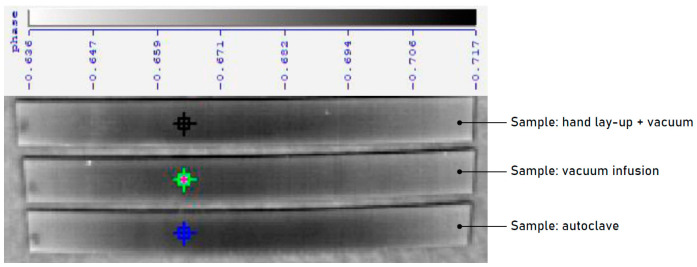
Samples produced with three different technologies during testing by active thermography method.

**Figure 3:**
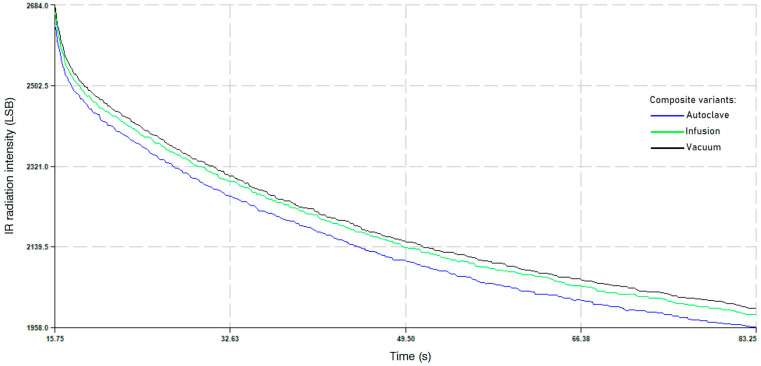
Results of tests conducted using active thermography for composites produced using three different technologies.

**Figure 4:**
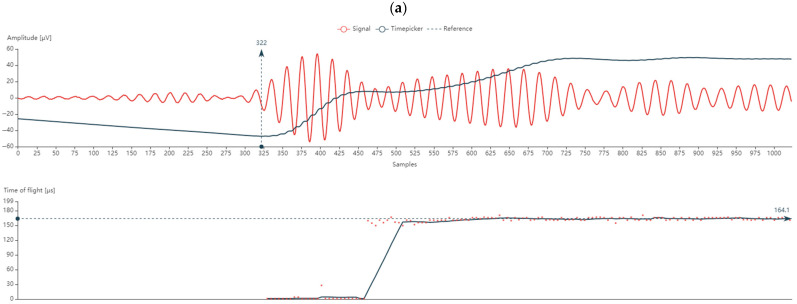

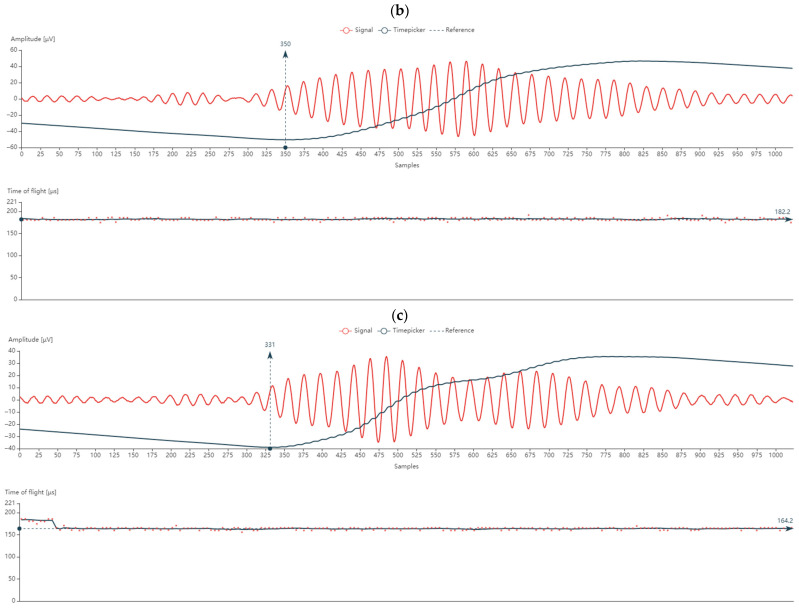
Results of the velocity of sound wave propagation in composite samples fabricated with: hand lay-up (**a**), vacuum infusion (**b**) and autoclave (**c**).

In order to verify the quality of the CF/E composites in terms of uniformity and lack of inclusions and air voids, NDTs were carried out by using the active thermography method for samples fabricated by the hand lay-up, vacuum infusion and autoclave processes. Active thermography is a non-destructive testing technique that uses the analysis of infrared images produced by active heating or cooling. The thermal excitation used in active thermography research may be sinusoidally variable, a unit step or Dirac pulse. The obtained thermogram sequences were analyzed using the discrete Fourier transform. The resulting phase images of all samples fabricated by different fabrication techniques were compared with each other. It was found that none of the CF/E composite samples had any features of structural discontinuity and no inclusions or air bubbles. However, differences were found in the density of composites produced using different fabrication processes. As expected, samples produced in an autoclave have the lowest density, while those produced using the vacuum method have the highest density. Figure 2 shows the samples tested using the described NDT method. Figure 3 presents results of tests conducted using active thermography. Research using active thermography allows determining the influence of the parameters/method of production on, among others, homogeneity of structure, density (heat capacity) and possible defects of CFRP composites. The course of the temperature change curves represented by changes in the intensity of the IR radiation signal during cooling, as a function of time (Figure 4), having the character of exponential functions decreasing and also uniform character without peaks or stops, indicates a homogeneous structure of CFRP composites, without defects in the form of pores, cracks or delaminations. At the same time, the differences in the cooling rate visible in the form of different slopes of the curves as a function of time indicate different densities (heat capacity) of the tested laminates, with the highest density being shown by the samples made by the hand lay-up + vacuum method (black curve), similar for the samples made by the vacuum infusion method (green curve), and the lowest for the samples made in an autoclave (blue curve).

Figure 4 shows results of the velocity of sound wave propagation in composite samples fabricated with hand lay-up, vacuum infusion and autoclave.

Based on the test results, it can be stated that the density is uniform or that there are disturbances in the volume structure of the composite. The most uniform in terms of the density of the structure is the composite produced by the infusion method, which is evidenced by the uniform signal course from its excitation because of the automatic suppression (Figure 4b). In turn, the most inhomogeneous density is shown by the composite produced by the hand lay-up method, which can be seen on the basis of the amplitude course of the excited signal, which significantly fluctuates before the final suppression (Figure 4a). On the other hand, the composite produced in the autoclave shows a slight inhomogeneity in the density of the structure (Figure 4c).

**Figure 5:**
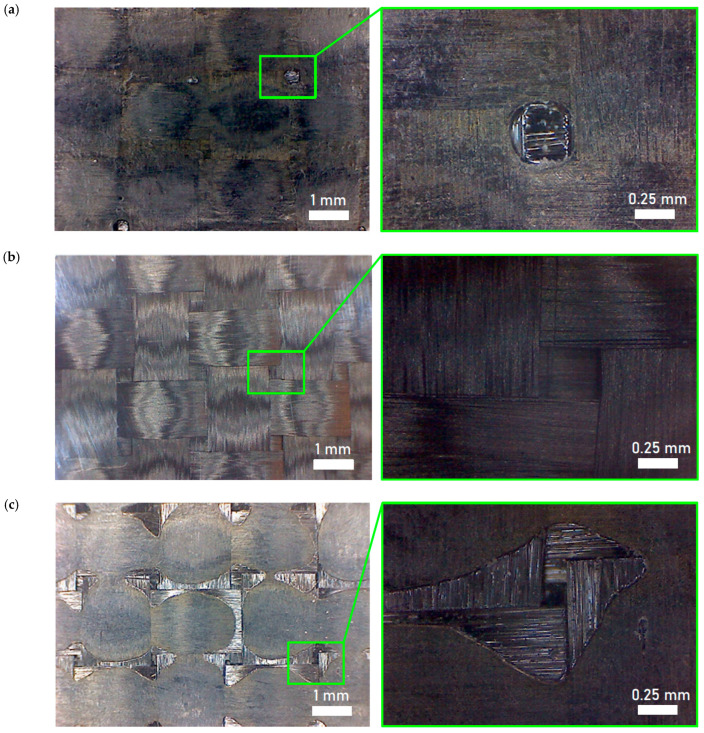
View of the composite surface at different magnifications for the variants: autoclave (**a**), infusion (**b**) and vacuum (**c**).

As part of the quality assessment of the composites, their surface was also analyzed before the destructive tests. The surface was inspected at a macroscopic magnification from the mold side, because no significant differences were observed from the release ply side. Figure 5 shows views of the composite surfaces for the individual variants at different magnifications. In the case of the composite produced in an autoclave (Figure 5a), a uniform surface covered with resin was demonstrated, however, sporadically in the places where there was interlacing of fibers in the fabric air bubbles with diameters ranging from 0.1 mm to 0.25 mm were observed. The highest surface quality is characterized by the composite produced by the infusion method (Figure 5b). In this case, the entire surface is covered with an even layer of resin, and no air bubbles were observed. In the case of the variant produced by the hand lay-up method (Figure 5c), inhomogeneous resin filling in the areas of the interlacing of the fabric was demonstrated over the entire surface.

The panels produced using the individual technologies were weighed to determine the weight proportions of reinforcement in relation to the matrix. The composite produced in an autoclave showed the highest reinforcement ratio, the ratio being 68.9:31.1 (reinforcement: matrix, wt.%). In the case of the infusion method, the ratio was 62.3:37.7. The lowest reinforcement ratio, as expected, was shown for the hand lay-up variant, the ratio in this case being 58.5:41.5.

### 3.2. Mechanical Properties of the CFRP Composites

**Figure 6:**
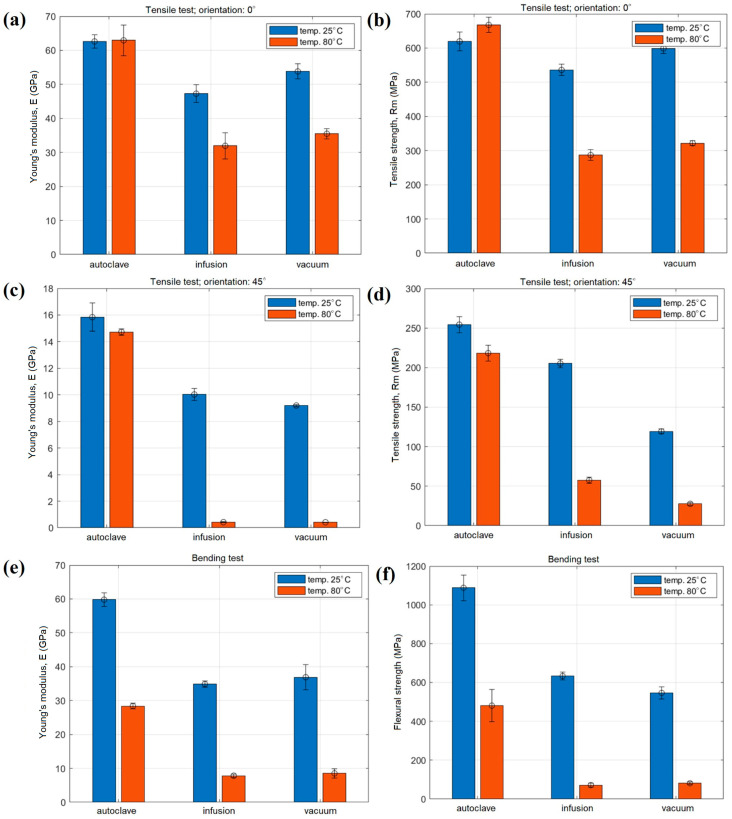
Results of static strength and Young’s modulus obtained in tensile test for sample orientation 0° (**a**,**b**) and 45° (**c**,**d**) and 3-point bending test (**e**,**f**) carried out at room and elevated temperature.

The mechanical properties of the CF/E composite were analyzed and are tabulated in Table 1 and Table 2. The tensile and flexural properties of the developed composite were analyzed by varying the fabrication process, orientation of the CF and testing temperature. The orientation of the CF was considered as 0° and 45° while fabricating the composites through different fabrication processes. Meanwhile, the testing temperature was employed as 25 °C or 80 °C. The maximum tensile strength and modulus were 668 MPa and 62.94 GPa, respectively. The maximum tensile properties were achieved by the autoclave fabrication process, 0° CF orientation and 80 °C testing temperature. Moreover, the ultimate flexural strength and modulus were recorded as 1088 MPa and 59.79 GPa, respectively. The maximum flexural properties were noticed in the autoclave fabrication process at 0° CF orientation and 25 °C testing temperature. Figure 6 shows the effects of fabrication process, testing temperature and fiber orientation on the tensile and flexural properties.

**Figure 7:**
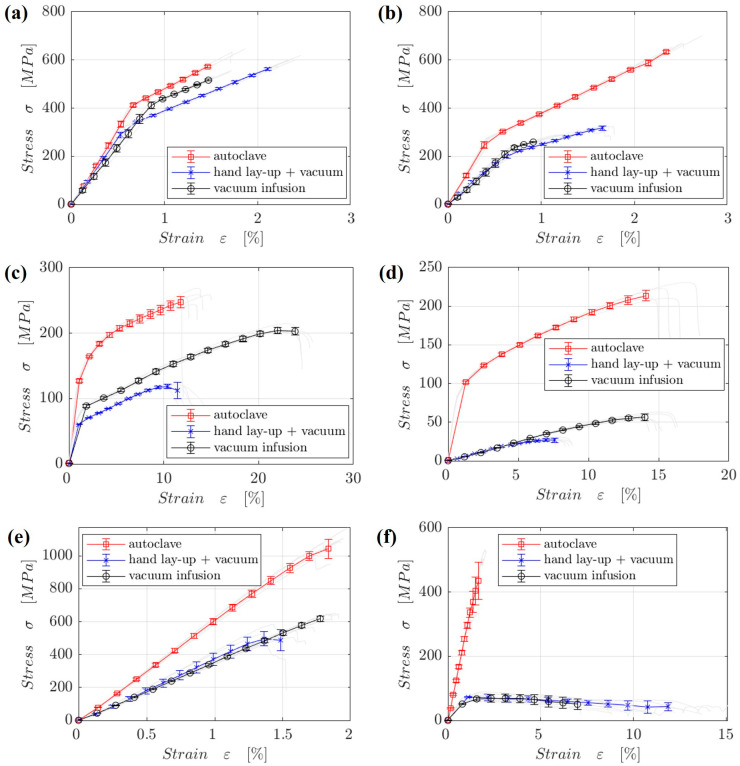
Comparison of curves obtained in the static tensile test of samples with an orientation of 0° carried out at 25 °C (**a**) and at 80 °C (**b**), with an orientation of 45° carried out at 25 °C (**c**) and at 80 °C (**d**) and bending test of samples carried out at 25 °C (**e**) and at 80 °C (**f**).

The specimen’s tensile modulus at 0° orientation is illustrated in Figure 6a. The Young’s modulus is maximum at 25 °C and 80 °C for the specimens that were autoclaved. Specimens made by infusion exhibit a moderate modulus, whilst those prepared by vacuum display the lowest values. The modulus declines for all methods at 80 °C, however, the autoclave shows a smaller reduction. Figure 6b displays the corresponding tensile strength, which, like Young’s modulus, is greatest for specimens treated with autoclave, then infusion and finally vacuum. Figure 6c,d shows that for a 45° orientation, the autoclave approach produces the strongest and most modulus- and strength-preserving results, while infusion produces results that are comparable but marginally weaker. At 80 °C, the modulus decreases significantly for all procedures, although the autoclave method keeps somewhat higher values. Figure 6e,f displays the flexural properties of the specimen. Similar to the tensile properties, the bending flexural strength and modulus are highest in autoclave-prepared specimens, followed by infusion and vacuum. All procedures exhibit a significant drop in modulus at 80 °C, although autoclave retains the material better. The use of high pressure and temperature during autoclave preparation results in composites with enhanced mechanical qualities, including tensile and flexural strength [26]. In addition to raising the modulus and mechanical strength, the high-pressure curing improves fiber alignment. A more stable fiber–matrix interface and improved resin crosslinking may be blamed for the lessened property decrease at 80 °C for the autoclave-fabricated specimen. The infusion process yields composites with average characteristics, which are better than those of vacuum methods but worse than those of autoclave. Compared to vacuum procedures, infusion guarantees good resin distribution and decreased void content; however, it falls short of autoclave operations in terms of compaction and fiber alignment, leading to worse mechanical qualities [27,28]. The matrix probably becomes more pliable at higher temperatures, leading to accelerated property deterioration. The mechanical characteristics are lowest in vacuum-prepared composites. Vacuum processing, due to its lower pressure, may increase void content and decrease fiber–matrix bonding efficiency. On the other hand, testing mechanical characteristics at 80 °C always results in a reduction. Even though epoxy resin is a thermoset, it can still remain soft at high temperatures, which decreases its strength and stiffness (modulus). However, because of improved crosslinking and reduced vacancies, specimens prepared by autoclave see a lessened matrix-softening effect. In the case of fiber orientation, the composite’s tensile and flexural strengths are often better when the fibers are oriented at 0° rather than 45°, since the fibers are more tightly packed in the direction of the load, providing more reinforcement. Figure 7 shows the stress–strain plot for the tensile and flexural properties. The autoclave specimens exhibit exceptional ductility and tensile strength at low temperatures, as shown in plots (a) and (c), where the greatest stress and strain values are observed for both the 0° and 45° orientations. Specimens prepared by manual lay-up in conjunction with vacuum infusion show the weakest ductility and strength, suggesting an increase in brittleness and poor fiber–matrix bonding due to the intrinsic brittleness of synthetic fibers [29]. The weakening of the polymer matrix at high temperatures is probably responsible for the decreased tensile strength and stiffness seen in plots (b) and (d) across all production processes. Nevertheless, specimens prepared in an autoclave continue to outperform the alternatives, exhibiting greater stress and strain values that attest to their exceptional thermal stability. Similar patterns in bending behavior are shown in plots (e) and (f). The specimens that are autoclaved exhibit excellent flexural properties, as they reach their maximum stress and strain at 25 °C (e). Although all methods exhibit a decrease in strength at 80 °C (f), the autoclave method remains comparatively strong even when subjected to high temperatures, as evidenced by the samples that retain relatively higher performance.

### 3.3. Delamination Analysis of the Composites

**Figure 8:**
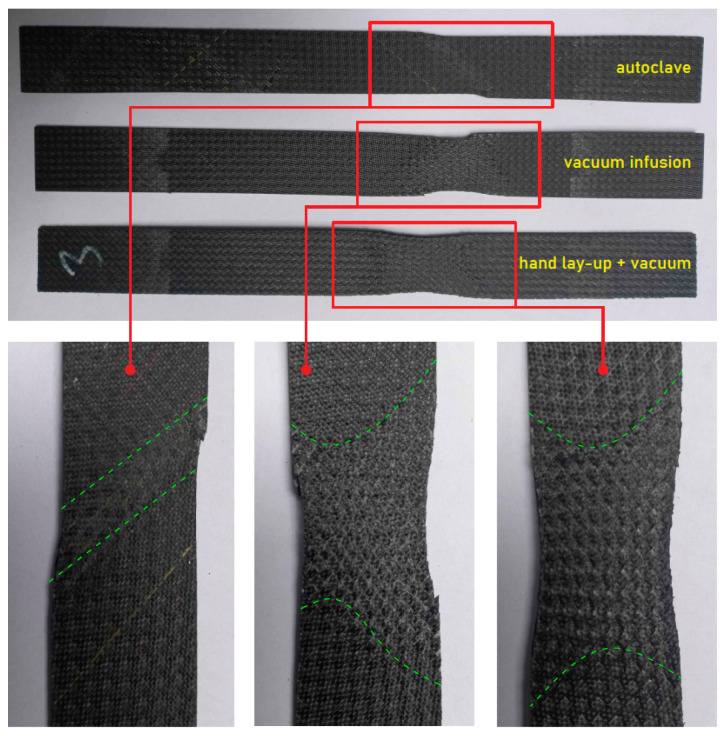
Different areas of delamination due to the failure of samples oriented at an angle of 45° and subjected to static tensile test.

**Figure 9:**
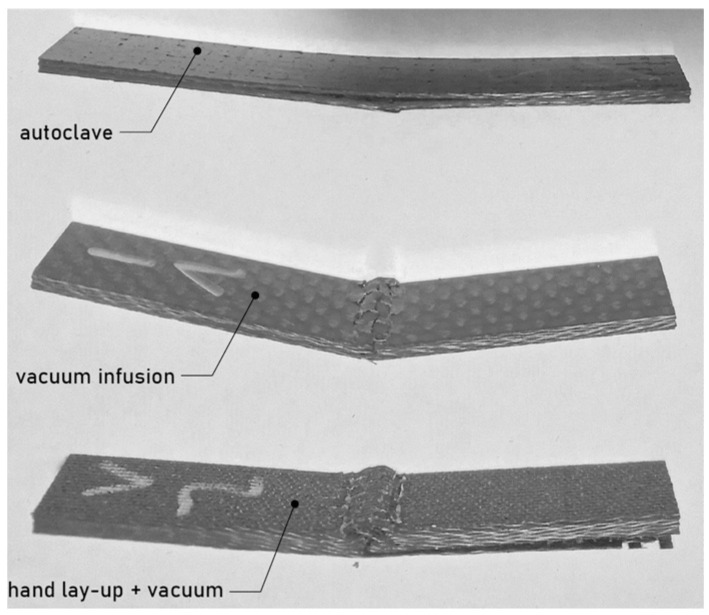
Different areas of delamination due to the failure of samples oriented at an angle of 45° and subjected to bending test.

Figure 8 and Figure 9 shows different areas of damage of specimens in tensile and flexural tests. In the case of samples oriented at an angle of 0°, they were completely separated during the static tensile test. However, in the case of samples oriented at an angle of 45°, after exceeding the strength limit the samples lost their load-bearing capacity but did not separate. Importantly, an equal mode of destruction was observed for each technology, showing delamination in a specific area, as shown in the figure below. The fracture in tensile-tested specimens manufactured using an autoclave seems clean and relatively acute. The fracture surface is smooth, indicating a brittle failure with minimal fiber pull-out or delamination. In comparison to autoclave specimens, vacuum infusion specimens have a less defined fracture zone. There are evident symptoms of matrix cracking and potential delamination. The surface has some roughness, indicating fiber pull-out and a weaker fiber–matrix bond. Hand lay-up specimens show considerably uneven failure, with substantial matrix cracking and fiber pull-out. The specimen has severe evidence of delamination, indicating poor fiber wet-out and bonding. In the instance of flexural testing, the autoclave specimen shows a progressive, smooth curvature, indicating a controlled failure mode. There is no considerable delamination on the surface, indicating a strong fiber–matrix link. The vacuum infusion specimen shows some buckling and delamination, especially at the bend points. The curvature is less smooth, suggesting an abrupt breakdown mechanism. There are apparent fissures and roughness on the surface, indicating a poorer connection than the autoclave specimen. The hand lay-up specimen shows considerable delamination and cracking. The surface is obviously rough and uneven, with clear evidence of fiber pull-out and matrix cracking. The autoclave technique gives the best mechanical performance due to the high pressure and temperature used during curing, which aids in resin impregnation and fiber consolidation [30]. This leads to fewer voids and better fiber–matrix adhesion. Tensile testing shows a clean, brittle fracture with smooth curvature. Vacuum infusion provides mediocre performance. The procedure offers good resin distribution but does not match the compact levels attained in autoclaving. This leads to some voids and weaker bonding, as indicated by the fiber pull-out and delamination observed in the tensile-tested specimens. In flexural testing, buckling and surface roughness indicate that the material cannot withstand as much stress as the autoclaved specimen without failing. The hand lay-up process with vacuum has the lowest performance due to manual resin application, which can result in uneven resin distribution and insufficient fiber wet-out. This leads to increased voids, weak spots and poor fiber–matrix interaction [31]. The extensive delamination, matrix cracking and fiber pull-out observed indicate that the bonding is insufficient to keep the fibers and matrix together during stress. These specimens fail catastrophically and suddenly indicating poor load-bearing capacity.

### 3.4. Elemental and Morphological Analysis

**Figure 10:**
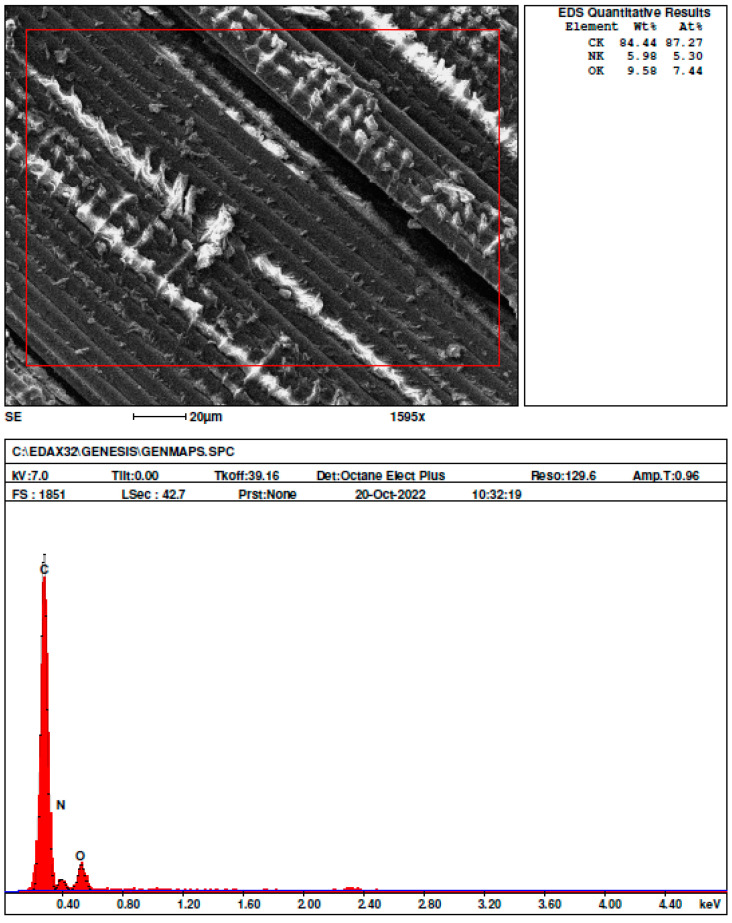
EDX analysis of composite specimen.

Elemental observations of the structure were carried out on the Nikon MA 200 optical microscope and on the SEM/FIB FEI Quanta 3D 200i. The microstructure of the fracture surface and the microstructure at the sites of sample delamination and fiber detachment from the matrix were analyzed. In selected samples, EDX chemical composition analysis was performed using the EDAX spectrometer as shown in Figure 10.

**Figure 11:**
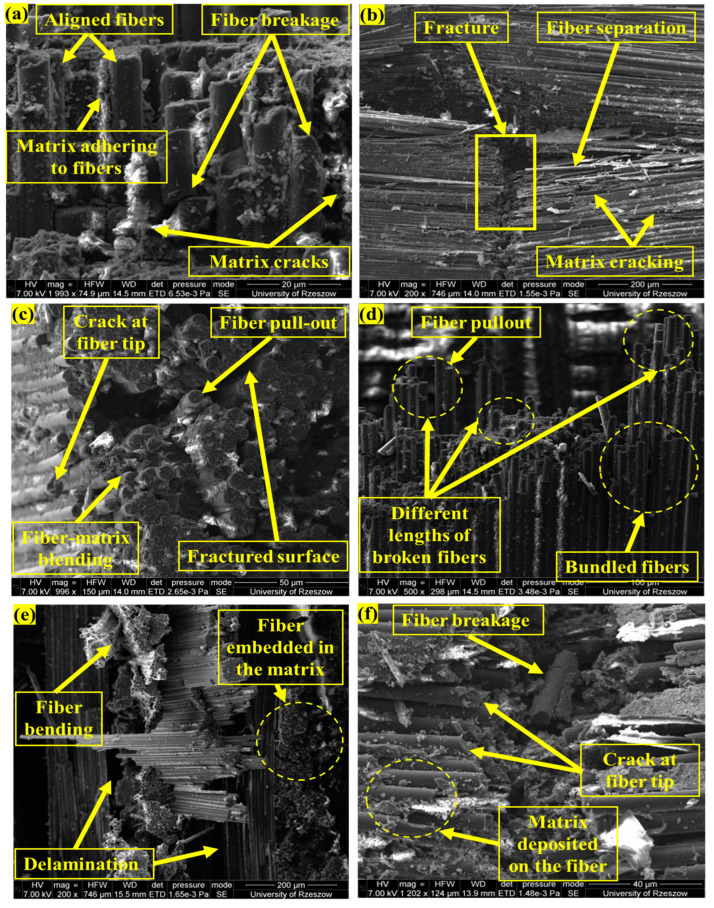
SEM images of mechanically tested specimen showing different modes of failure: fiber breakage in stress concentration areas (**a)**; parallel cracking of both fibers and matrix (**b)**; cracked surface with fiber pull-out (**c**); Fiber pull-out from the matrix and their breakage in various planes (**d**); fibers partially embedded in the matrix, with visible matrix breaking and delamination (**e**); matrix cracking and fiber fracture simultaneously (**f**).

A magnified view of the composite’s surface structure, as seen through a scanning electron microscope (SEM) picture, shows the carbon fibers’ arrangement. There is obvious roughness and a few surface flaws, and the fibers seem to be aligned; these may be caused by the fibers’ interactions with the matrix material. With an atomic percentage (At%) of 87.27% and a weight percentage (Wt.%) of 84.44%, the EDX analysis shows that carbon (C) is the dominant element, as observed through red spikes in Figure 10. The utilization of carbon fibers as the composite’s reinforcing element is supported by its high carbon content. The nitrogen (N) content is 5.98% by weight and 5.30% by absolute volume. The existence of oxygen (O)-containing functional groups is shown by the oxygen content, which is 9.58% Wt.% and 7.44% At%. This could indicate that the polymer matrix components or the fiber surface have undergone oxidation. Figure 10 shows the SEM images of the fractured specimen. Figure 11a depicts fiber ends that appear to have been neatly broken, with some matrix material still attached to the fibers. The fibers are aligned, and the matrix shows evidence of microcracking. Clean fiber fractures and matrix cracking indicate tensile stress, which causes fiber breakage at high-tension locations. Clean fractures in the fibers indicate a brittle fracture from tension. Cracks in the matrix may also indicate that the matrix failed before or at the same time as the fibers due to tension in the matrix phase [32]. Figure 11b shows a clean fracture that includes both fiber breaking and matrix cracking. The fracture surface looks to be rather smooth, with few fibers sticking out. Clean splits in the fibers and matrix show brittle failure. The matrix has cracked along the fracture surface, lining up with the fibers. Figure 11c depicts a rough, cracked surface with some fiber pull-out. The composite seems to have had a more disorderly failure. The existence of fiber pull-out shows that the fiber–matrix contact is weaker than the fibers’ tensile strength [33]. Large cracks in the matrix indicate that matrix failure occurred before fiber failure. Figure 11d demonstrates a thick fiber packing with obvious pull-out. The fibers appear fragmented at varying lengths, indicating a complicated failure mechanism. The varied fiber lengths and fiber pull-out indicate uneven stress distribution, which is a symptom of flexural loading [34]. Figure 11e shows fibers partially embedded in the matrix, with visible matrix breaking and delamination. Matrix cracking indicates that the matrix broke owing to stress on the convex side of the bend. Layer separation is a classic defect caused by interlaminar shear forces. Figure 11f also shows matrix cracking and fiber fracture, indicating that both the matrix and the fibers failed simultaneously.

### 3.5. DSC Analysis

**Figure 12:**
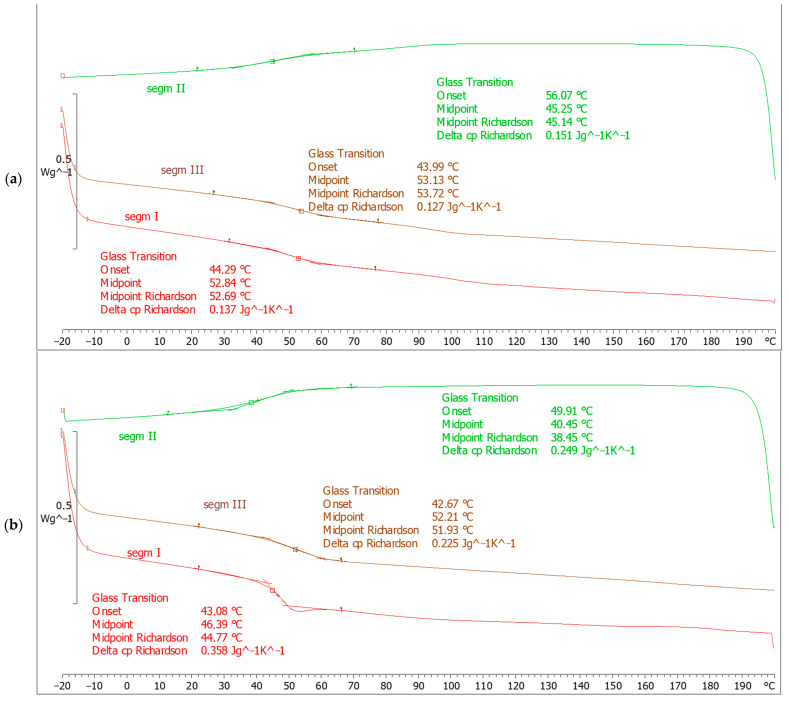
DSC analysis results of epoxy resins constituting the matrix of composites produced by hand lay-up (**a**) and infusion (**b**) methods.

Due to the significant decrease in mechanical properties of samples made using the infusion and hand lay-up methods under the influence of elevated temperature, DSC analysis was performed. Measurements were taken for cured resins constituting the matrix of the above-mentioned composite variants. Due to the fact that the samples made in the autoclave are based on pre-impregnation, no resin from this variant was available for comparative analysis. However, due to the specificity of the high-temperature autoclave process, composites are designed to work at elevated temperatures. Figure 12a shows a diagram of the DSC analysis for the Havel L285 MGS epoxy resin constituting the matrix of the composite made using the hand lay-up method. In this case, the glass transition temperature was shown to be 56.07 °C. It should be noted that, just like the composite in this variant, the resin subjected to DSC analysis was post-cured at 40 °C. Figure 12b shows the DSC analysis diagram for Havel LH288 epoxy resin, which was used as a matrix in the composite produced by the infusion method. A lower value of the glass transition temperature, i.e., 49.91 °C, was shown here. In the case of both analyzed variants, the glass transition temperature is significantly lower than the elevated temperature of +80 °C, at which the strength tests were carried out. Therefore, the properties of the resins are undoubtedly the cause of the significant decrease in fatigue properties at elevated temperatures. The supplier of both considered resins provides information that it is possible to increase the range of mechanical property retention by post-curing at +80 °C for 90 min. Therefore, further research is planned in the future, which will be aimed at verifying the possible degree of stability of mechanical properties at elevated temperatures.

### 3.6. Fatigue Lifetime Analysis

**Figure 13:**
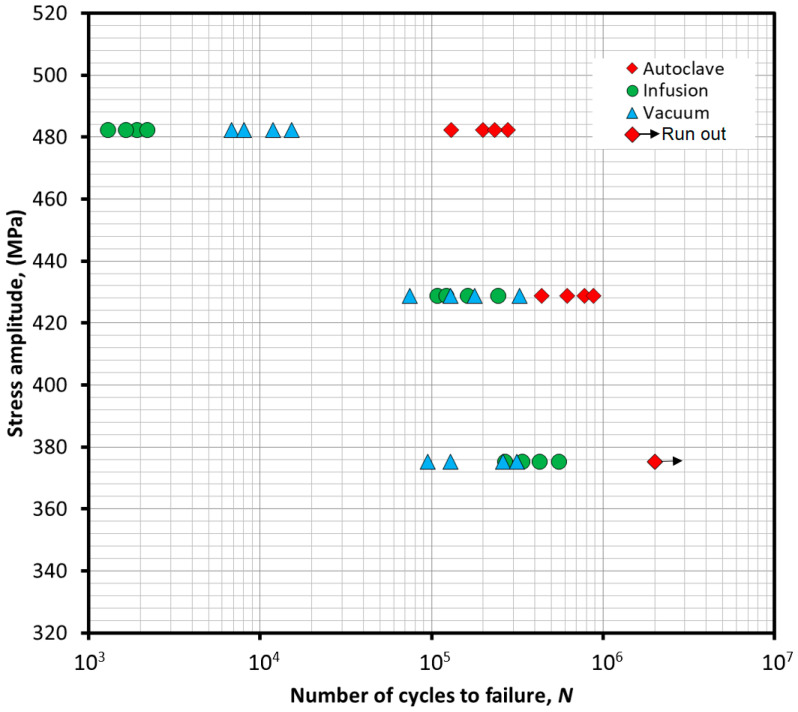
Comparative fatigue diagram for the tested composite variants.

Comparative studies of fatigue properties of composites manufactured using different technologies were carried out at three different levels of cyclic loading. When determining the load level, the lowest average value of static tensile strength in the direction of 0° stretching at a temperature of +25 °C was assumed as the reference value. The lowest static strength was demonstrated for samples manufactured by the infusion method, for which the average value was 536 MPa. Cyclic load levels of 90%, 80% and 70% of this value were assumed. Comparative studies were carried out for all variants considered at the indicated levels of variable loading. Table 3 summarizes the results of fatigue tests indicating the fatigue lifetime for subsequent samples at individual levels of cyclic loading. In turn, Figure 13 presents a fatigue graph for all variants.

At the highest of the considered cyclic load levels, all composite samples produced by the infusion method were destroyed in the area conventionally assumed as low-cycle fatigue. The average fatigue lifetime for this variant was 1.762 × 10^3^ cycles with a standard deviation of 336.1. For composites produced by the hand lay-up method, the fatigue lifetime was on average 10.536 × 10^3^, which is 497.8% longer than the infusion variant. A significantly longer fatigue lifetime was demonstrated for composites produced in an autoclave, in this case the average value was 210.406 × 10^3^. This is a longer fatigue lifetime by 11837.9% than the vacuum variant and by 1897.1% than the hand lay-up variant. Moving on to the load level constituting 80% of the static tensile strength of composites produced by the infusion method, it was shown that the average fatigue lifetime values for the infusion and vacuum variants are similar, amounting to 159.539 × 10^3^ cycles and 176.620 × 10^3^ cycles, respectively. However, for composites produced by the infusion method, greater repeatability was shown, the standard deviation in this case is 53.401 × 10^3^, while for the hand lay-up variant it is 93.062 × 10^3^. At the considered level of cyclic load, the average fatigue lifetime for samples produced in an autoclave was 678.788 × 10^3^ with a standard deviation of 167.204 × 10^3^. At the lowest load level considered, which is 70% of the tensile strength of the infusion variant, it was shown that composite samples produced in an autoclave do not undergo fatigue failure after reaching the assumed limit number of cycles equal to 2 × 10^6^ and, on a macroscopic scale, no signs of destruction were shown for these samples after the tests. On the other hand, for samples produced by the infusion method, an average fatigue lifetime of 397.505 × 10^3^ cycles was shown with a standard deviation of 106.892 × 10^3^. At this level of fatigue load, the infusion variant is characterized by a longer average fatigue lifetime than the vacuum variant by 99.2%. For the latter variant, the average fatigue lifetime was 199.524 × 10^3^ cycles with a standard deviation of 90.138 × 10^3^.

Summing up the results of comparative fatigue tests, it should be noted that samples produced in an autoclave are characterized by significantly higher fatigue properties than the other variants. Although the division into low- and high-cycle fatigue applies to metals due to the conventional limit of plastic deformation during fatigue loading, the discussion will refer to this division for illustration purposes, for which the limit is usually assumed at around 10^4^ cycles. At the highest fatigue load level considered, referring to the aforementioned division into low- and high-cycle fatigue, it can be observed that samples for the infusion and vacuum variants are destroyed in the low-cycle fatigue area. In turn, samples produced in the autoclave process fail in the high-cycle fatigue area. In turn, at the lowest fatigue load level of 375.2 MPa, samples produced in the autoclave demonstrate fatigue strength at the assumed limit number of cycles of 2 × 10^6^. On the other hand, samples for the infusion and vacuum variants have demonstrated fatigue lifetime below 400 × 10^3^ cycles. A more detailed comparison should be made for the infusion and vacuum variants. At the highest fatigue load level, significantly higher durability was demonstrated for connections produced by the hand lay-up method. However, at lower levels of variable load in the area conventionally assumed as high-cycle fatigue, the trend reverses. At the lowest level of cyclic load, the average fatigue lifetime for the infusion variant is twice as high as for the vacuum variant. Considering the trend shown, it can be concluded that the fatigue strength for the infusion variant would be significantly higher than for the vacuum variant.

It was also shown that the fatigue test results for the infusion variant are characterized by greater repeatability than for the vacuum variant.

Both the longer fatigue lifetime at lower load levels and the greater repeatability of the results can be explained by the greater homogeneity of the structure and surface of the composite panels manufactured by the infusion method. The hand lay-up method, due to the manual application of the resin, may be characterized by a certain degree of inhomogeneity, which may result, among others, from the skills and experience of the person performing the lamination process.

**Figure 14:**
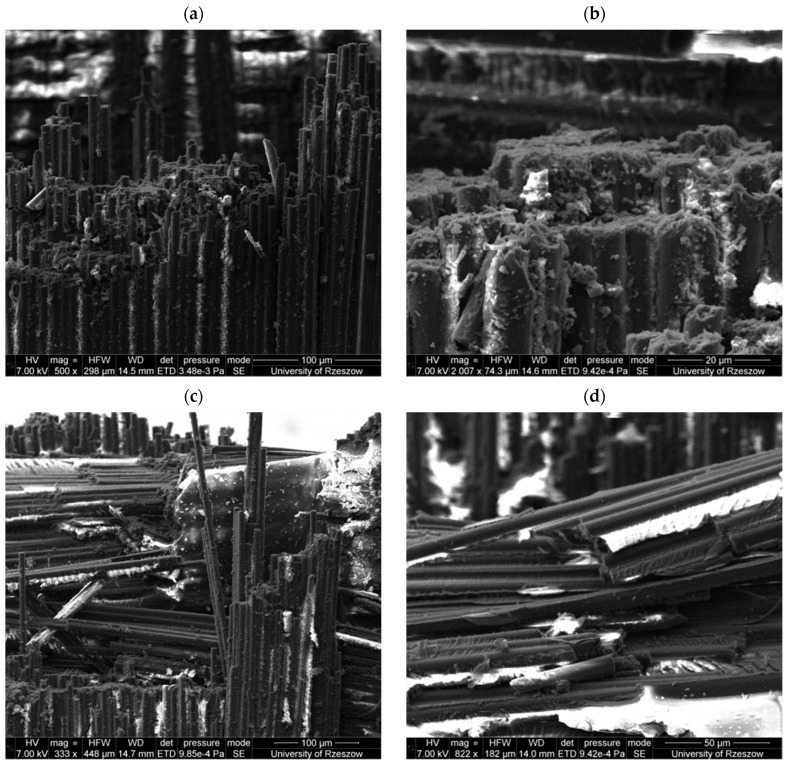
Fatigue fractures for samples of the following variants: autoclave (**a**), infusion (**b**) and vacuum at different magnifications (**c**,**d**).

Moving on to the analysis of fatigue fractures, a comparison was made for samples that showed a similar level of fatigue life. In the case of infusion, a sample was taken into account that was destroyed after 268.127 × 10^3^ fatigue cycles at a cyclic load of a maximum value of 375.2 MPa (Figure 14a). In this case, the fibers were broken in the destruction area, with visible shear planes of the composite layers. However, no significant separation of individual fibers from the matrix was observed over a larger area. It was also observed that in the shear area, resin fragments remained on the fiber surfaces, which indicates proper adhesion between the fibers and the resin. Moving on to the composite variant produced in an autoclave, a sample that was destroyed after 278.061 × 10^3^ fatigue load cycles at a variable stress level of 482.4 MPa (Figure 14b) was subjected to fractographic analysis. In this case, the nature of the destruction is very similar to that of the infusion variant, most of the fibers were torn at a similar level due to stretching and shear planes are visible between the composite layers. Similarly, high adhesion between the fibers and the resin was demonstrated here, as evidenced by the resin residues on the fiber surfaces in the matrix shear planes. A different situation was demonstrated for the vacuum variant, in this case a sample with a fatigue life of 261.039 × 10^3^ was analyzed at a cyclic load of 375.2 MPa. In this case, larger delamination areas were observed, as well as individual separated fibers (Figure 14c). It was also shown that the fibers torn from the matrix exhibit large areas free of resin (Figure 14d), which may indicate relatively low adhesive strength between the fibers and the resin.

## 4. Conclusions

In this study, carbon fiber/epoxy composites were fabricated with three different technologies, i.e., hand lay-up, vacuum infusion and autoclave techniques. The fabricated samples were tested for their mechanical properties, namely tensile and flexural strength, by varying the fiber orientation and testing temperature. Delamination analysis and morphological analysis were also performed for the fractured specimen. The following conclusion can be drawn from this study.

Autoclave-fabricated composites outperform vacuum infusion and hand lay-up composites in terms of tensile and flexural characteristics. This approach results in more effective fiber alignment and higher compaction, which contribute to the enhanced stiffness and strength seen in the stress–strain study. None of the composites showed any sign of structural discontinuity, inclusions or air bubbles during the non-destructive testing of the composite specimen.

The highest tensile strength and modulus were obtained at 668 MPa and 62.94 GPa for autoclave specimens fabricated at 0° fiber orientation and tested at 80 °C. Similarly, for samples with 0° fiber orientation and at a testing temperature of 25 °C, the highest flexural strength was observed. Testing at elevated temperatures (80 °C) reveals a loss in mechanical characteristics across all manufacturing processes due to the softening of the polymer matrix. However, autoclave-prepared composites show a lesser loss in characteristics, indicating a more stable fiber–matrix interface and increased resin crosslinking. Due to the significant decrease in mechanical properties at elevated temperature, DSC analyses were performed for epoxy resins constituting the matrices of the infusion and vacuum variants. In both cases, it was shown that the glass transition temperature is lower than +80 °C and is 49.91 °C and 56.07 °C for the infusion and vacuum variants, respectively.

SEM images reveal that autoclave-processed specimens have a smoother and more uniform surface morphology, with fewer voids and flaws than those prepared using vacuum procedures. The EDS data show the presence of a well-distributed matrix, indicating that resin impregnation and fiber bonding were successful in autoclave specimens.

Delamination studies show that autoclave-prepared composites have the lowest tendency to delaminate due to greater fiber–matrix adhesion and reduced void content. Delamination in vacuum-prepared specimens indicates reduced interfacial bonding, compromising the composite’s structural integrity.

Comparative fatigue tests have shown that the fatigue life at a maximum cyclic load of 428.8 MPa is comparable for the vacuum and infusion variants, the average value being 176.620 × 10^3^ cycles and 159.539 × 10^3^ cycles, respectively. In turn, significantly longer fatigue life is demonstrated by the composite produced in an autoclave, for which the average fatigue life value was 678.788 × 10^3^.

In summary, the presented comparison of selected mechanical properties of CFRP composites confirms the generally known knowledge that composites manufactured in an autoclave are characterized by the highest quality in terms of mechanical properties (among the considered technologies). However, this is the most energy-intensive technology, and composites manufactured in an autoclave based on prepregs are significantly more expensive in relation to other technologies. Therefore, the economic effect will ultimately be the decisive factor in choosing the manufacturing technology for given design solutions.

## Figures and Tables

**Table 1:** Tensile properties of the composites tested.

Sample Description. (Manufacture Method; Orientation: Testing Temperature)	Young’s Modulus			Tensile Stress R_m_, MPa			Elongation A, %		
	E. (GPa)	std E. (GPa)	CV E. %	R_m_. (MPa)	std R_m_. (MPa)	CV R_m_. %	ε. [-]	std ε. [-]	CV ε. %
Autoclave; 0°; +80 °C	62.942	4.461	7.09	668	22	3.34	0.0250	0.0014	5.8
Autoclave; 0°; +25 °C	62.606	1.957	3.13	619	27	4.38	0.0170	0.0015	8.57
Autoclave; 45°; +80 °C	14.731	0.236	1.60	218	10	4.64	0.1557	0.0111	7.13
Autoclave; 45°; +25 °C	15.836	1.059	6.69	254	10	4.03	0.1309	0.0115	8.77
Infusion; 0°; +80 °C	31.926	3.810	11.93	287	16	5.56	0.0120	0.0015	12.20
Infusion; 0°; +25 °C	47.255	2.623	5.55	536	16	3.06	0.0160	0.0010	6.47
Infusion; 45°; +80 °C	0.417	0.035	8.45	58	4	7.08	0.1418	0.0078	5.49
Infusion; 45°; +25 °C	10.030	0.452	4.51	205	5	2.55	0.2293	0.0045	1.97
Vacuum; 0°; +80 °C	35.478	1.548	4.36	322	7	2.27	0.0168	0.0009	5.19
Vacuum; 0°; +25 °C	53.799	2.222	4.13	599	15	2.49	0.0236	0.0009	4.00
Vacuum; 45°; +80 °C	0.412	0.018	4.37	28	2	6.62	0.0747	0.0039	5.22
Vacuum; 45°; +25 °C	9.197	0.083	0.90	119	3	2.64	0.1055	0.0057	5.42

**Table 2:** Flexural properties of the composites in tests.

Sample Description. (Manufacture Method; Orientation; Testing Temperature)	Flexural Modulus			Flexural Stress R_m_, MPa			Elongation A, %		
	E. (GPa)	std E. (GPa)	CV E. %	R_m_. (MPa)	std R_m_. (MPa)	CV R_m_. %	ε. [-]	std ε. [-]	CV ε. %
Autoclave; 0°; +80 °C	28.380	0.861	3.03	481	83	17.31	0.0188	0.0019	9.97
Autoclave; 0°; +25 °C	59.796	2.081	3.48	1088	66	6.05	0.0189	0.0009	4.87
Infusion; 0°; +80 °C	7.843	0.522	6.66	71	10	14.58	0.0251	0.0097	38.74
Infusion; 0°; +25 °C	34.861	0.932	2.67	634	21	3.29	0.0186	0.0004	1.93
Vacuum; 0°; +80 °C	8.522	1.390	16.31	81	7	8.04	0.0148	0.0037	24.88
Vacuum; 0°; +25 °C	36.894	3.770	10.22	547	31	5.67	0.0153	0.0014	9.06

**Table 3:** Fatigue test results for individual samples.

Level of Cyclic Load (MPa), R = 0.1, f = 25 Hz	Composite Variant		
	Autoclave	Infusion	Vacuum
	Number of Cycles to Failure N × 10^3^ for Individual Samples		
482.4	129.743	1.291	6.843
	278.061	1.908	15.327
	199.632	1.647	8.039
	234.187	2.204	11.933
428.8	437.851	108.596	129.073
	619.345	162.548	74.386
	778.329	245.369	324.698
	879.627	121.645	178.324
375.2	2000 (run out)	427.106	129.278
	2000 (run out)	339.897	261.039
	2000 (run out)	268.127	312.845
	2000 (run out)	554.891	94.933

## Data Availability

The original contributions presented in this study are included in the article. Further inquiries can be directed to the corresponding author.
